# NLRP3 Inflammasome From Bench to Bedside: New Perspectives for Triple Negative Breast Cancer

**DOI:** 10.3389/fonc.2020.01587

**Published:** 2020-09-04

**Authors:** Margherita Sonnessa, Antonella Cioffi, Oronzo Brunetti, Nicola Silvestris, Francesco A. Zito, Concetta Saponaro, Anita Mangia

**Affiliations:** ^1^Functional Biomorphology Laboratory, IRCCS Istituto Tumori “Giovanni Paolo II”, Bari, Italy; ^2^Medical Oncology Unit, IRCCS Istituto Tumori “Giovanni Paolo II”, Bari, Italy; ^3^Department of Biomedical Sciences and Human Oncology, University of Bari Aldo Moro, Bari, Italy; ^4^Pathology Department, IRCCS Istituto Tumori “Giovanni Paolo II”, Bari, Italy

**Keywords:** inflammasome, NLRP3, microenvironment, breast cancer, TNBC

## Abstract

The tumor microenvironment (TME) is crucial in cancer onset, progression and response to treatment. It is characterized by an intricate interaction of immune cells and cytokines involved in tumor development. Among these, inflammasomes are oligomeric molecular platforms and play a key role in inflammatory response and immunity. Inflammasome activation is initiated upon triggering of pattern recognition receptors (Toll-like receptors, NOD-like receptors, and Absent in melanoma like receptors), on the surface of immune cells with the recruitment of caspase-1 by an adaptor apoptosis-associated speck-like protein. This structure leads to the activation of the pro-inflammatory cytokines interleukin (IL)-1β and IL-18 and participates in different biological processes exerting its effects. To date, the Nod–Like Receptor Protein 3 (NLRP3) inflammasome has been well studied and its involvement has been established in different cancer diseases. In this review, we discuss the structure, biology and mechanisms of inflammasomes with a special focus on the specific role of NLRP3 in breast cancer (BC) and in the sub-group of triple negative BC. The NLRP3 inflammasome and its down-stream pathways could be considered novel potential tumor biomarkers and could open new frontiers in BC treatment.

## Introduction

Cancer is a multifactorial disease in which the tumor microenvironment (TME) plays a central role through the release of inflammatory cytokines, chemokines and growth factors, which are major components in inflammation and cancer (1, 2). The TME activates innate immune cells that may act as oncogenes or oncosuppressors, depending on the activation of inflammatory molecules and the cancer type, site, or stage. Inflammation is a crucial factor of cancer development and progression (3–6). Thus, clarifying the molecular mechanisms implicated in the complex interaction between malignancy and chronic inflammation is critical for cancer prevention and management. Different mechanisms have been recognized to play a key role in cancer associated inflammation pathways. In particular, the involvement of inflammasomes in cancer has attracted growing attention over the past few years, leading to new potential cancer treatment strategies.

The inflammasome is the main component of the innate immune system and its assembly is triggered by many endogenous and exogenous signals. Inflammasome activation leads to the maturation and secretion of pro-inflammatory cytokines, such as interleukin (IL)-1β, and IL-18 (7–9). Appropriate regulation of inflammasome activation is essential since its dysfunction results in different diseases and cancer (10).

A better understanding of the relationship between inflammasomes and tumorigenesis could provide promising and effective approaches against breast cancer (BC), especially in the subset of triple negative breast cancer (TNBC), which are neoplasms with an aggressive profile and still short of specific therapeutic options. In this review, we provide basic information about inflammasomes and highlight how the Nod–Like Receptor Protein 3 (NLRP3) inflammasome and its downstream pathways influence the pathogenesis and progression of BC, and in particular, of TNBC.

### Inflammasome Sensors: Pathogen Recognition Receptors Superfamily

Inflammasome activation is initiated upon triggering of the superfamily of Pathogen Recognition Receptors (PRRs), which are components of immune cells capable of detecting pathogens and include Toll-like receptors (TLRs), NOD-like receptors (NLRs), and Absent in melanoma like receptors (ALR) (11).

### Toll-Like Receptors

Toll-like receptors are *trans*-membrane receptors usually expressed in immune and epithelial cells. They respond to exogenous stimuli known as pathogen-associated molecular patterns (PAMPs) and danger-associated molecular patterns (DAMPs) (12). Recent studies have shown increased messenger RNA (mRNA) levels of TLR3, TLR4, and TLR9 in BC (13). Elevated levels of TLRs expression are associated to high recurrence in BC patients. In particular, tumors with high TLR3, and TLR4 expression have been associated with a greater probability of metastasis (13, 14). Although Jukkola-Vuorinen and colleagues demonstrated TLR9 expression in the epithelial tissue of most of their BC patients, with higher levels in negative estrogen receptor (ER) than in positive ER tumors (15), the significance of TLR9 remains controversial. Reduced TLRs expression seems to be involved in decreased cell proliferation and survival, suggesting that these could be possible therapeutic targets (16).

### NOD-Like Receptors

NOD-like receptors are intercellular receptors that are able to identify PAMPs and DAMPs, and thus activate the innate immune response. NLRs have a tripartite structure consisting of a carboxy-terminal leucine-rich repeat domain (LRR), a central nucleotide-binding oligomerization domain [NBD or NOD, also known as nucleotide-binding and oligomerization (NACHT) domain] and a variable N-terminal protein-protein interaction domain. In humans, there are 22 known NLRs divided into 4 functional categories: inflammasome assembly, signaling transduction, transcription activation, and autophagy.

The variable N-terminal region of NLRs consists of a Pyrin domain (PYD) or a caspase activation and recruitment domain (CARD) which interact with the nucleotide binding and oligomerization domain. Deregulation of these receptors may lead to different diseases and be also involved in apoptosis and in early cancer development (7).

### Inflammasomes: Structure and Mechanisms of Action

**FIGURE 1:**
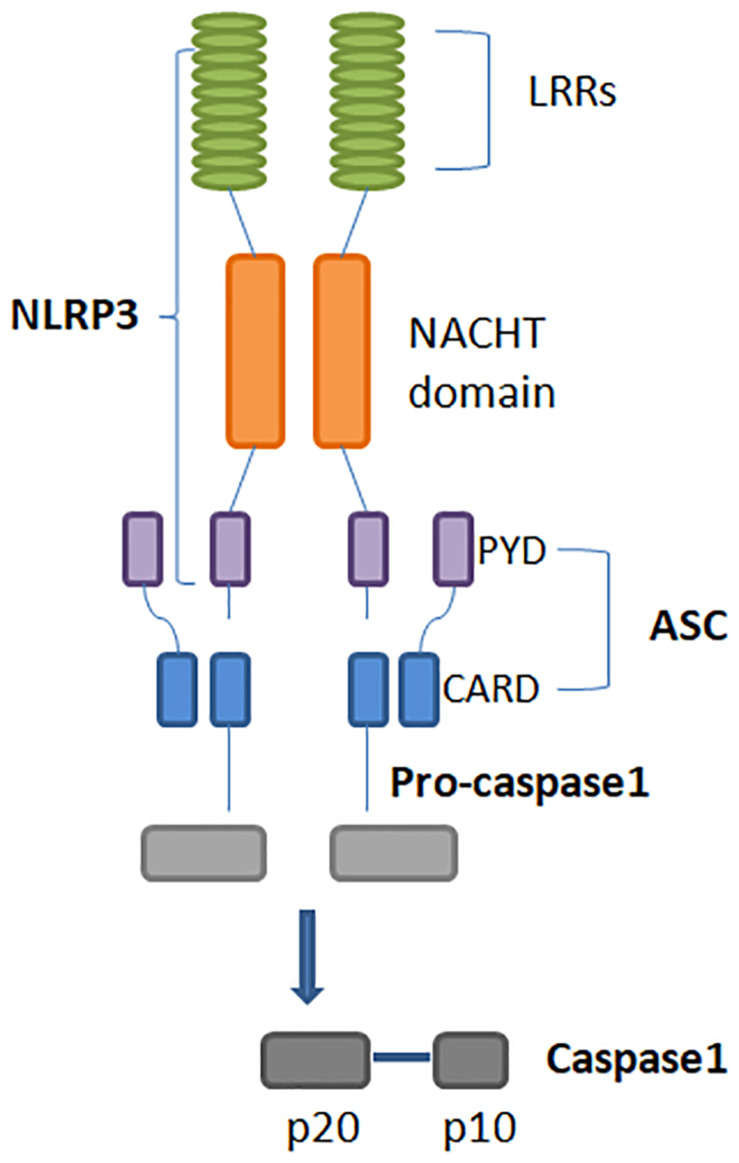
Structure of the NLRP3 inflammasome. Its activation leads to homotypic interaction between the Pyrin domains of NLRP3 and ASC. ASC is required for recruitment of pro-caspase-1. Interaction between ASC and pro-caspase-1 CARD domains result in the active form of caspase-1, that is composed of large and small subunits, p20 and p10. NLRP3: Nucleotide-binding domain (NOD)-like receptor protein 3. ASC: Apoptosis-associated speck-like protein containing a CARD.

Inflammasomes are multimeric protein complexes that typically comprise a sensor for PAMPs and DAMPs, an adaptor molecule known as apoptosis-associated speck-like protein containing a CARD (ASC) and a pro-caspase. ASC is also identified as PYCARD and it is characterized by two domains: Pyrin and CARD (Figure 1). Homotypic CARD-CARD or PYD-PYD interactions are required for inflammasome assembly and interaction with caspases. Once activated, caspases generate mature forms of IL-1β or IL-18, and gasdermin D (GSDMD). GSDMD mediates a form of inflammatory cell death called pyroptosis (7). Inflammasomes are classified as canonical and non-canonical (Table 1). Canonical inflammasomes include NLRP3, NLRC4, NLRP1, NLRP6, and NLRP12, which function as platforms for caspase-1 (CASP-1) activation (17). Non-canonical inflammasomes include murine CASP-11 and its human homologues, such as CASP-4, and CASP-5 inflammasomes (18).

**TABLE 1:** Canonical and Non-Canonical Inflammasomes.

Canonical Inflammasome	Structure	Function
NLRP1	– N-terminal PYD – Central NBD – LRR – FIIND – C-terminal CARD	Recruiting and activation of CASP-1 by CARD domain
NLRC4	– N-terminal CARD domain – Central NBD – C-terminal LRR domain	Recruiting and activation of CASP-1 and caspase- 8 by CARD domain
NLRP6	– PYD – NBD – LRR	Recruiting of ASC by PYD-PYD interaction, triggering its polymerization
NLRP12 (Monarch-I or PYPAF7)	– PYD – NBD – LRR	Inhibition of canonical and non-canonical NF-κB pathway Activation of CASP-1

**Non-Canonical Inflammasome**	**Structure**	**Function**

Caspase -4/-5/-11	– CARD – Large subunit – Small subunit	Activated by LPS. Oligomerization and activation by LPS and CARD domain binding. Promoting pyroptosis and secretion of pro-IL-1β and pro-IL-18 via the canonical NLRP3 inflammasome activity.

*CARD, Caspase activating and recruitment domain; FIIND, “Function to find” domain; LPS, Lipopolysaccharides; LRR, Short leucine-rich repeat domain; NBD, Nucleotide-binding domain; NLRC4, Nucleotide-binding domain and leucine-rich repeat containing 4; NLRP1, NOD-like receptor family pyrin domain containing 1; NLRP6, NOD-like receptor family pyrin domain containing 6; NLRP12, Leucine-rich repeat and PYD-containing protein 12; pro-IL-1β, pro-interleukin-1β; pro-IL-18, pro-interleukin-18; and PYD, Pyrin domain.*

### Nod–Like Receptor Protein 3 Inflammasome

**FIGURE 2:**
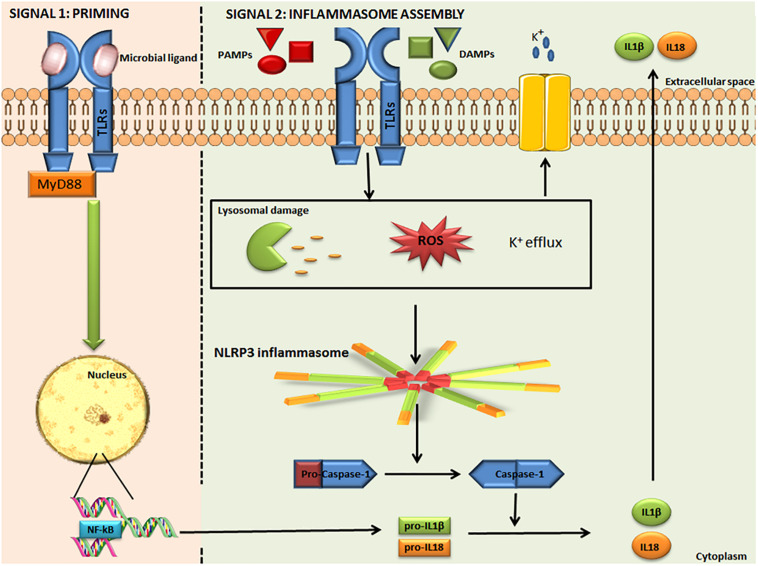
Activation of the canonical NLRP3 inflammasome. The NLRP3 inflammasome is activated in two steps. Signal 1: microbial ligands are recognized by TLR that leads to NF-κB pathway activation and pro-IL-1β and pro-IL-18 up-regulation. Signal 2: involves different PAMPs, DAMPs, ATP, and particulates that promote lysosomal damage, mitochondrial ROS production or K^+^ efflux, resulting in NLRP3 inflammasome complex assembly and activation. The NLRP3 inflammasome interacts with pro-caspase-1 and promotes its conversion into caspase-1, which catalyzes the proteolytic cleavage of pro-IL-1β and pro-IL-18, resulting in active IL-1β and IL-18. IL-1β and IL-18 are released into the extracellular space. NLRP3: Nucleotide-binding domain (NOD)-like receptor protein 3. TLR, Toll like receptor; NF-κB, nuclear factor-kappa Beta; pro-IL-1β, pro-interleukin 1 beta; pro-IL-18, pro-interleukin 18; PAMPs, pathogen-associated molecular patterns; DAMPs, danger-associated molecular patterns; and ROS, Reactive Oxygen Species. Some parts of this figure are from SMART Service Medical Art (Smart.servier.com).

Nod–Like Receptor Protein 3 is the most well-studied inflammasome, expressed mainly by myeloid lineage cells. The NLRP3 inflammasome is an intracellular protein complex, with a tripartite domain structure including LRR, NBD, and PYD (Figure 1). ASC is required for NLRP3 assembly and recruitment of CASP1. Two distinct steps are necessary for NLRP3 activation: priming and inflammasome assembly. Priming involves microbial ligands recognized by TLRs and activates the nuclear factor kappa-light-chain-enhancer of activated B cells (NF-κB) pathway, resulting in an up-regulation of NLRP3 expression. The assembly step may entail different stimuli, leading to activation of the NLRP3 inflammasome complex. When the inflammasome binds pro-CASP-1, this enzyme is cleaved into p10 and p35 fragments which are subsequently processed into CARD and a p20 subunit. The assembly of the p10 fragment with 2 molecules of p20 will finally form the active CASP-1 enzyme, which operates by activating IL-1β and IL-18 (Figure 2) (19, 20). The main models for NLRP3 activation are potassium (K^+^) efflux with lysosomal destabilization and reactive oxygen species (ROS) production. The first mechanism causes a reduction in intracellular K^+^ by different pathogens that can secrete toxins able to generate pores and increase K^+^ efflux, resulting in NLRP3 inflammasome activation (21, 22). In the second mechanism, NLRP3 oligomerization is also driven by ROS generation, as a consequence of mitochondrial dysfunction (23).

### Regulation of NLRP3 Inflammasome Expression and Activity

The NLRP3 inflammasome is involved in the immune response to infections, regulating the secretion and activation of inflammatory cytokines and promoting pyroptosis. Overexpression of the NLRP3 inflammasome can cause excessive inflammation, associated with the pathogenesis of different autoimmune, chronic inflammatory and metabolic diseases, such as diabetes, gout, and cardiovascular/neurological disorders. So far, effective regulation of NLRP3 activation is required to help prevent all these disorders (24). An important checkpoint involved in NLRP3 and pro-IL-1β expression is NF-κB. It is located in the cytoplasm in an inactive form. Its translocation and activation in the nucleus of inflammatory cells results in angiogenesis and in increased growth and survival of cancer cells. The activation of NF-κB is associated to elevated expression levels of cell-cycle genes (such as cyclin D1), inhibitors of apoptosis, and proteases that promote the invasive phenotype (25).

## Inflammasomes and Their Controversial Role in Cancer

Inflammasome activation is a mechanism affecting multiple crucial cell processes such as proliferation, death, metabolic pathways, inflammatory signaling and immune reactions (10). Therefore, inflammasomes constitute a key factor both in physiological and pathological conditions.

But what role do they play in the development of diseases? Although their protective function in infectious diseases and their contribution to the development of autoimmune disorders are well known, the role of inflammasomes in tumor growth remains controversial, leaving several unclear aspects, including their involvement in biological processes. Inflammasomes can promote carcinogenesis by enhancing a pro-cancer TME or, instead, they can exert anticancer activity by regulating pyroptosis and the immune system. The final effects are influenced by the different types of inflammasomes implicated, the compartments in which inflammasomes are activated, the heterogeneity of cancer cells and the microenvironment, the type of tumor and the different chemokines and cytokines involved (26, 27).

Interleukin-1β and IL-18 are the major cytokines activated by inflammasomes and having an immunosuppressive effect. High concentrations of these cytokines have been observed in tumor tissues (28–30). In particular, IL-1β promotes carcinogenesis, tumor proliferation, and invasion (31). Increased IL-1β levels in the tumor environment have been related to a worse outcome of patients (32–34), supporting its central role in inflammation-associated tumor progression. However, it has been shown that inflammasomes are implicated in different types of cancers with distinct roles, depending on the context and on the tumor type (35, 36). Recent “*in vivo*” studies have demonstrated that components of the inflammasome complex can have a protective role in induced colon cancer. Such a bivalent behavior could be explained by a synergic effect of different mechanisms, such as activation of the immune response, balance of oncogenic, and oncosuppressive factors and defective apoptosis (35–37). NLRP3 is expressed in cells from the myeloid lineage, such as monocytes, macrophages, myeloid-derived suppressor cells (MDSCs), and Tumor-associated macrophages (TAMs), which may have a dual role during tumorigenesis. They can promote cancer through the release of pro-inflammatory mediators creating an immunosuppressive microenvironment, allowing cancer cells to escape immunosurveillance, and supporting tumor progression. On the other hand, macrophages are also involved in the antitumor response through activation of anti-proliferative effects (38).

Liu and colleagues described a dual role of ASC also in different phases of melanoma. ASC is involved in the inhibition of the NF-kB pathway and melanomagenesis in primary melanoma, while it activates the NF-kB pathway and supports cancer progression in metastatic melanoma through a loop of IL-1β-dependent NF-kB autoactivation (39).

Inflammasome activity can also be influenced by other variables, such as the gastrointestinal microbiota, contributing to the progression of some diseases (40, 41).

Once again, the TME plays a fundamental role in tilting the balance toward supporting or inhibiting tumor development.

### Gastrointestinal Tract Cancers

Different studies have described the role of inflammasome components in colorectal cancer (CRC), but their exact roles may be associated to the context of cancer development. PYCARD, CASP-1, and NLRP3 protect against against tumorigenesis as shown in studies that demonstrated increased tumor growth in mice lacking NLRP3 inflammasome components (35). The NLRP3 inflammasome enhances IL-18 expression, a liver metastasis inhibitor, by inducing the tumoricidal activity of Natural Killer (NK) cells (37). The NLRP3 inflammasome stimulates IL-1β secretion that promotes CRC cell proliferation and invasion by inducing epithelial-mesenchymal transition (42). However, the results regarding the role of inflammasome components in CRC development produced in several studies remain controversial. The role of the inflammasome is clearly much more complex and the intricacies in defining it are generally not only related to methodological differences (i.e., antibodies, animal model, molecular assays, etc.). Its specific role depends on several factors, including expression pathways, effector/inhibitor molecules, tumor types, and stages. Further, the balance of TME molecules, pro and anti-angiogenic factors (VEGF, HIF-1α, and IL-12), and immune cells (cancer-associated fibroblasts, tumor-infiltrating immune cells, and TAMs) and the expression of receptors of the innate immune system (TLRs, NLRs) can influence the function of inflammasomes (36, 43, 44).

In gastric cancer (GC), there is significant evidence substantiating the association between chronic inflammation, inflammasomes and tumor development. It has been proven that *Helicobacter Pylori* (*H. Pylori*) is an important signal for NLRP3 inflammasome activation and consequent IL-1β and IL-18 secretion (45). *H. Pylori* infection leads to gastric inflammation with the activation of cyclin-D1 and triggering of the NF-kB signaling pathway, resulting in tumor proliferation, invasion, and metastasis (46). Low CASP-1 expression is correlated with tumor stage, metastasis and patient survival (47). Another study revealed increased expression of ASC and IL-18 in GC, suggesting a pro-tumorigenic effect for ASC by preserving cells against apoptosis (48). These findings indicate that GC may result from the activation of different pathways dependent on the NLRP3 inflammasome and make the latter a potential therapeutic target, although further investigations are required to confirm such a possibility (49).

Inflammasomes play contrasting roles in liver cancer. NLRP3 inflammasome constituents are significantly down-regulated in human hepatocellular carcinoma (HCC) as compared to inflamed and normal hepatic tissues (50). Sex hormones have been shown to decrease HCC progression by increasing NLRP3 inflammasome activation. An “*in vitro*” study has revealed that of NLRP3 was up-regulated by 17β-estradiol (E2), a principal form of estrogen, through the E2/ERβ/MAPK (mitogen-activated protein kinase) pathway (51). By contrast, other studies suggest that the NLRP3 inflammasome has an opposite function. Under hypoxic conditions, the High Mobility Group Box 1 protein (HMGB1) has been observed to activate the NLRP3 inflammasome, thus elevating CASP-1, IL-1β, and IL-18 levels, and promoting HCC invasion and metastasis (52). Hence, inflammasome components, especially IL-1β and IL-18, play an important role in HCC development and progression (53–56).

### Other Cancers

In melanoma, the NLRP3 inflammasome components, CASP-1, IL-1β, and IL-18, are involved in tumorigenesis and metastasis formation (36, 57–60). Differences in ASC expression can also promote or inhibit tumor development via NF-κB activity (39). However, the effect of the inflammasome on melanoma mostly depends on the tumor grade and the tissue in which the inflammasome is activated (60).

Inflammasome components have a damaging role in lung cancer. Recent studies have shown that, although it is absent in Melanoma 2 (AIM2), the inflammasome is overexpressed in non-small cell lung cancer (NSCLC). On the other hand, the NLRP3 inflammasome has been shown to be up-regulated in high-grade adenocarcinoma (ADC) and small cell lung cancer. CASP-1, IL-1β, and IL-18 are overexpressed in NSCLC and ADC and their expression is associated with chemoresistance (61). In addition, the NLRP3 inflammasome has been observed to sustain cancer progression and lymph node metastasis in NSCLC patients by reducing E-cadherin and increasing Snail through IL-1β secretion (62). These studies have demonstrated that inflammasomes support tumorigenesis and cell proliferation in lung cancer by releasing inflammatory cytokines and decreasing immune function.

In recent years, a link has been highlighted to exist between inflammation and prostate cancer (PC). Hypoxia, one of the major impulses for PC development, has a role in activating NLRP3 and AIM2 inflammasomes (63). In turn, the AIM2 inflammasome stimulates prostate hyperplasia and cancer development (64).

Nod–Like Receptor Protein 3 inflammasome activation has been found to sustain the development of glioblastoma and was predictive of poor survival in a cohort of radiotherapy-treated patients. These findings are supported by evidence that NLRP3 inhibition decreased tumor proliferation and improved survival in murine models (65).

Finally, the NLRP3 inflammasome has a key role in promoting progression and invasion of head and neck squamous cell carcinoma, as evidenced by studies showing that NLRP3 inflammasome components are over-expressed in squamous cell carcinoma tissues (66).

In sum, the detrimental effects of inflammasomes in most cancers are probably due to excessive activation of inflammasomes in the TME. Given the intricate roles of inflammasome signaling, it is indispensable to develop specific anticancer therapies for various tumor types.

## Inflammasomes and BC

**FIGURE 3:**
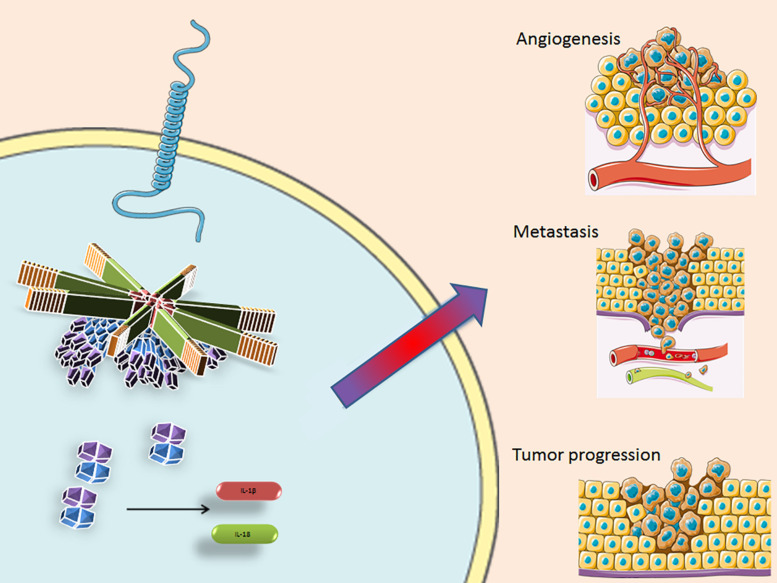
The inflammasome facilitates pro-caspase-1 recruitment by the ASC protein, that cleaves and converts it into its activate form. Casp-1 then activates IL-1β and Il-18, amplifying the inflammatory response and influencing the TME, supporting angiogenesis, metastasis formation and tumor progression. NLRP3, Nucleotide-binding domain (NOD)-like receptor protein 3; ASC, Apoptosis-associated speck-like protein containing a CARD; TLR, Toll like receptor; IL-1β, interleukin 1 beta; and IL-18, interleukin 18. Some parts of this figure are from SMART Service Medical Art (Smart.servier.com).

During the past few years, it has been hypothesized that different inflammasomes may be involved in the development of BC. Several studies have shown that the AIM2 inflammasome has a peculiar protective role in BC in that it enhances apoptosis (67) and suppresses cancer cell proliferation by inhibiting NF-kB activity (68). NLRP3 inflammasome activation and IL-1β secretion play a critical role in promoting tumor growth and metastasis in BC (43) and they are associated with tumor proliferation, angiogenesis, invasiveness, relapse and progression (69–72) (Figure 3). A relationship between local levels of IL-1β and mammary malignancy was observed in a murine model of BC (73). A recent study demonstrated that CASP-1 activation leads to the production of matrix Metalloproteinase-9 (MMP-9), which is involved in metastasis development in radiotherapy-resistant BC (74). NLRP3 also leads to a reduction in the antitumor immunity of T and NK cells, thus creating an inflammatory microenvironment supporting BC progression and metastasis by activating inflammatory signaling pathways, such as the NF-κB/STAT 1/3, and IL-1β/IL-1RI/β-catenin pathways (43, 75). NLRP3 stimulates the enrolment of myeloid cells into the TME, especially of MDSC and TAMs, thereby enhancing cancer development. Moreover, NLRP3 activation and IL-1β production are associated with tumor lymphangiogenesis and lung metastasis in BC (36).

Zhang and colleagues showed that microRNA-223-3p (miR-223-3p) had an inhibiting effect on the NLRP3 pathways (76). Previous studies had already considered that MiR-223-3p may be involved in the regulation of inflammatory response, immunosuppression, cell growth and angiogenesis in different cancer cells (77, 78). MiR-223-3p mimics suppressed NLRP3 expression, leading to an increased apoptotic rate and reduced proliferative capacity and ASC, IL-1β, and IL-18 expression levels. Consistent with “*in vitro*” results, “*in vivo*” experiments demonstrated that miR-223-3p reduced tumor growth and increased survival rate in mice (76). Collectively, these data indicate that targeting the inflammasome/IL-1 pathway with a miR-223-3p-like molecule could suppress the growth of BC cells by inactivation of the NLRP3 inflammasome. Hence, the role of inflammasomes is closely related to the TME via the activation of different biological pathways. From bench to bedside, this role is crucial for the patients’ health, especially in some conditions directly implicated in chronic inflammation such as autoimmune diseases and obesity. It is known that obesity is a risk factor for the development of different types of cancer, in particular BC. Kolb et al. showed a connection between obesity-associated NLRC4 inflammasome activation and IL-1β secretion related to angiogenesis and BC progression (79). In a recent study, NLRP3 was shown to be involved in leptin-induced BC. Leptin is a hormone secreted by adipocytes that supports BC cells migration by activating the NLRP3 inflammasome and enhancing IL-18 expression (80, 81). Activation of the inflammasome has also been found to increase BC cell growth. Globular adiponectin has been shown to decrease BC cell growth as a result of NLRP3 inflammasome inhibition (82). An *in vitro*/*in vivo* study showed that BRCA1 deficit affected mitochondrial function, mitophagy and NLRP3 inflammasome activation, thus promoting metastasis (83). The findings of these studies provide evidence that inflammasome inhibition could serve as a therapeutic target for the treatment of BC.

Two of the principal limitations of the studies regarding inflammasome are: (a) the absence of clinical data supporting “*in vivo*” and “*in vitro*” experimental evidence and (b) the actual assessment methods used in daily practice at Pathology Departments. These two aspects are closely related given the lack of consistent protocols and clinically acknowledged methods for diagnosis, which makes any application to clinical practice challenging. In our opinion, immunohistochemistry (IHC) could be an easy, rapid and inexpensive method to obtain information about the inflammatory state of a tumor. IHC would be fast and it would fit into the routine diagnostic protocols already in place in Pathology Departments. Determining a patient’s inflammatory state could be an additional piece in the diagnostic puzzle of cancer that can help identify a group of patients with a worse prognosis. However, additional studies designed to define inflammasome activity in different tumor types are warranted before effective recommendations can be made in this field.

## TNBC: an Open Challenge in Preclinical Studies

Triple negative breast cancers show clinical and genetic features that make effective treatment arduous. To date, there are no targeted therapies available for patients with advanced TNBC (84). This subgroup of tumors represents a very heterogeneous disease, consisting of a wide spectrum of biologically distinct subtypes with different prognosis (85). TNBC harbors a specific inflammatory microenvironment in which high levels of molecules released from activated inflammatory cells, such as pro-inflammatory cytokines, ROS, reactive nitrogen species (RNS), coexist with angiogenic factors, such as the Vascular Endothelial Growth Factor (VEGF), and with a significant amount of Tumor-Infiltrating Lymphocytes and TAMs (86). This inflammatory TME increases the risk of cancer, tumor invasion and metastases. TNBC are more aggressive than others phenotypes and have a particularly poor prognosis. A thorough understanding of the biology of TNBC becomes crucial to discover new molecular targets and biomarkers that can support drug development and clinical decision-making, thus improving the survival and quality of life of these patients (87, 88).

In particular, inflammasomes open up new therapeutic perspectives for these cancer types. Several “*in vitro*” and “*in vivo*” studies have been performed to examine the role of inflammasomes in TNBC and explore potential novel targets for treatment (Table 2). A recent study investigated the role of docosahexaenoic acid (DHA) in triggering the signaling pathway of pyroptosis in a TNBC cell line model characterized by high aggressiveness, invasiveness and poor differentiation (89). DHA is an omega-3 fatty acid with anticancer activity that inhibits cell proliferation by increasing apoptosis (90–93) and reducing cell invasiveness (94). DHA has also been shown to decrease BC progression and lung metastasis by suppressing MPs (95). Pizato and colleagues demonstrated that DHA induced pyroptosis-associated markers, such as NF-κB, HMGB1, CASP-1, GSDMD, and IL-1β, resulting in cancer cell death. These Authors also highlighted that DHA is cytotoxic only for cancer cells, whereas it has no significant effect on human non-cancerous mammary epithelial cells (89).

**TABLE 2:** Inflammasome and TNBCs: “*in vitro/in vivo*” studies.

Authors [References]	Molecule	Results
Pizato N. et al. (89)	Docosahexaenoic acid (DHA)	Cell death by pyroptosis
Yao M. et al. (96)	Barberine (BBR)	Reduction of cellular viability Modulation of NLRP3 inflammasome signaling
Han J. et al. (71)	Zerumbone (ZER)	Anti-inflammatory effect Reduction of cellular migration and invasion
Huang G. C. et al. (100)	Zerumbone (ZER)	Inhibition of cell growth Induction of DNA fragmentation Prolongation of the life of mice.
Si L. et al. (101)	Silibinin	Inhibition of the NLRP3 inflammasome pathway by decrease of mitochondrial activity and ROS generation
Byun H. J. et al. (103)	Silibinin	Inhibition of cellular proliferation, migration and invasion
Wang T. et al. (104)	Nanoparticles of hydrophilic arsenic sulfide (e-As4S4)	Inhibition of NLRP3 expression by down-regulation of ROS levels and reduction of HIF-1α expression

*NLRP3, Nod-like receptor protein-3; ROS, Reactive Oxygen Species; and HIF-1α, Hypoxia-inducible factors-1α.*

Another recent study has shown that barberine (BBR), a natural alkaloid isolated from Chinese herbal plants, reduces the viability of TNBC cells and increases lactate dehydrogenase (LDH) release, suppressing colony formation and migration of TNBC cells. BBR was also observed to reduce the secretion of pro-inflammatory cytokines implicated in carcinogenesis, especially IL-1β, IL-1α, IL-6, and tumor necrosis factor alpha (TNF-α), and to modulate NLRP3 inflammasome signaling by down-regulating mRNA and the protein expression of its related gene (96).

In another study, Han J. et al. investigated the effect of Zerumbone (ZER) on IL-1β-induced cell migration and invasion in a TNBC cell line (71). ZER is a sequiterpene isolated from Southeast Asian ginger having antioxidant properties and capable of inhibiting tumor cell growth and promoting apoptosis (97–100). ZER was shown to have an anti-inflammatory effect on the IL-1β signaling pathway by inhibiting the downstream expression of IL-8 and MMP-3, which play a pivotal role in invasion and migration of tumor cells. Therefore, ZER could indirectly suppress IL-1β- induced migration and invasion of TNBC cells (71).

A very recent “*in vitro*” study tested the effects of silibinin on a TNBC cell line (101). Silibinin is a natural polyphenolic flavonoid with anticancer effects. It induces autophagy in human BC cells (102) and inhibits proliferation, migration and invasion of TNBC cells via the inhibition of the Jak2/STAT3/MMP2 signaling pathway (103). This study demonstrated that silibinin impaired mitochondrial activity and ROS generation, leading to inhibition of both the NLRP3 inflammasome pathway and CASP-1/IL-1β expression, reducing cell migration and invasion (101).

Finally, a new study applied nanoparticles of hydrophilic arsenic sulfide (e-As4S4) to a TNBC murine model to investigate its impact in this type of tumor (104). A bioavailable form of arsenic sulfide, e-As4S4 has already been shown to have a therapeutic effect in leukemia (105, 106). There is evidence that e-As4S4 reduces ROS production, thus avoiding one of the major causes of inflammasome triggering. Its capability to regulate ROS makes it an attractive strategy for TNBC treatment. Arsenic accumulation in tumor tissues was observed to cause down-regulation of ROS levels and a significant reduction in hypoxia-inducible factors-1α (HIF-1α) expression, leading to the inhibition of NLRP3 expression. This recent study showed that tumor metastases to the lungs and liver declined and survival of the TNBC mice was prolonged (104).

All these findings have proved the direct or indirect anticancer effect of different molecules on TNBC models and focused on the regulation of the NLRP3 inflammasome pathway as a novel and promising strategy for anti-TNBC treatment, pointing to its potential therapeutic role for clinical use.

### Future Prospects

Inflammasomes are involved in numerous chronic inflammation-related diseases, including cancer, thus garnering increasing interest in investigations regarding their place in clinical practice. In particular, the NLRP3 inflammasome has been implicated in cancer development, given its ability to activate the pro-inflammatory cytokines IL-1β and IL-18 which cause metabolic, inflammatory, hematological, and immune effects. Inflammasome factors may have oncogenic effects in specific types of cancer, including BC, and may therefore represent novel diagnostic and therapeutic targets for this disease. However, their specific role in TNBC is very complex and has not yet been well defined. Further, more extensive studies are required to envision their use in clinical settings in the future. TNBC treatment is an urgent need worldwide, because of the increasing incidence of this disease, its early onset, rapid progression, and high metastatic potential and very poor prognosis. Chemotherapy is still considered the main treatment modality for TNBC, but it often leads to resistance to conventional therapies (107). To date, there exists substantial evidence of a strong relationship between inflammasome-activated pathways and TNBC and inflammatory cytokines and other inflammasome component have been shown to be aberrantly expressed in this cancer subtype.

It is interesting to note that TNBC displays immunogenic characteristics with a rich immune infiltrate that makes the use of immune checkpoint inhibitors (ICIs) attractive for this cancer in the neo-adjuvant, adjuvant, and metastatic settings (108). Inflammasomes could be crucial to predict the response to ICI treatment or they may even become one of the strategies to improve ICI anticancer efficacy (109). In an “*ex vivo”/“in vitro”* study, PD-1 inhibition was observed to lead to CD8 + T cell activation through a PD-L1-NLRP3 inflammasome signaling cascade. This event induced recruitment of MDSCs into cancer tissues, with a reduction in anticancer response. The inhibition of NLRP3 was seen to decrease MDSC tumor infiltration thereby increasing the efficacy of anti-PD-1 antibody immunotherapy. Thus, “anti-inflammasome” treatment could represent a promising strategy against TNBC (110).

## Conclusion

In conclusion, the distinctive molecular hallmarks displayed by this aggressive type of cancer could represent potential objectives for the development of new target drugs and personalized treatments which could greatly enhance the management of TNBC.

## Author Contributions

MS and AC performed the literature search, acquired, and collated the data. MS, AC, and CS drafted the manuscript. CS prepared the figures. OB, NS, and FZ critically revised the manuscript. AM conceived, designed, and coordinated the manuscript. All authors read and approved the final manuscript.

## Conflict of Interest

The authors declare that the research was conducted in the absence of any commercial or financial relationships that could be construed as a potential conflict of interest.
